# Evidence that dog ownership protects against the onset of disability in an older community-dwelling Japanese population

**DOI:** 10.1371/journal.pone.0263791

**Published:** 2022-02-23

**Authors:** Yu Taniguchi, Satoshi Seino, Bruce Headey, Toshiki Hata, Tomoko Ikeuchi, Takumi Abe, Shoji Shinkai, Akihiko Kitamura

**Affiliations:** 1 Japan Environment and Children’s Study Programme Office, National Institute for Environmental Studies, Tsukuba, Japan; 2 Research Team for Social Participation and Community Health, Tokyo Metropolitan Institute of Gerontology, Tokyo, Japan; 3 Melbourne Institute of Applied Economic and Social Research, The University of Melbourne, Melbourne, Australia; 4 Human Care Research Team, Tokyo Metropolitan Institute of Gerontology, Tokyo, Japan; 5 Faculty of Nutrition, Kagawa Nutrition University, Saitama, Japan; Ehime University Graduate School of Medicine, JAPAN

## Abstract

**Objectives:**

This study examined the association between dog and cat ownership, the onset of disability and all-cause mortality in an older population. Dog and cat owners take more regular exercise and have closer social relationships than non-owners. We further assess the beneficial effects of these moderating variables on the onset of disability and mortality.

**Methods:**

Dog and cat ownership data were collected from 11233 community-dwelling adults age 65 years and older. These data were matched with data about the onset of disability held by the Japanese long-term care insurance system. Local registry data were used to ascertain all-cause mortality.

**Results:**

During the approximately 3.5 year follow-up period, 17.1% of the sample suffered onset of disability, and 5.2% died. Logistic regression analysis indicated that, compared with a reference group of those who had never owned a dog (odds ratio fixed at 1.0), older adults who were currently dog owners had a significantly lower odds ratio of onset of disability (OR = 0.54 95% CI: 0.37–0.79). Our results further show that regular exercise interacts with dog ownership to reduce the risk of disability. The association of dog and/or cat ownership with all-cause mortality was not statistically significant.

**Conclusions:**

Dog ownership appears to protect against incident disability among older Japanese adults. Additional benefits are gained from ownership combined with regular exercise. Daily dog care may have an important role to play in health promotion and successful aging.

## Introduction

In the last century and a half, a major achievement of medical science has been greatly extended life expectancy. In the wake of this achievement, successful aging and healthy aging have become important goals and are usually defined as the absence of disease and disability [1, 2]. To achieve successful aging, health promotion policy needs to focus on maintaining functional capacity without disability for as long as possible during the life span.

Previous studies have reported that physical frailty greatly increases the risk of future disability [3–5]. A recent study indicated that older adults who were frail at baseline had 4 times the hazard ratio for incident disability compared with robust older adults; this during a 2-year follow-up period [3]. Frailty prevention is important for avoiding disability and so for successful and healthy aging.

There is now an accumulating body of knowledge on risk factors that can be modified to reduce the risk of disability [6–8]. In recent studies we reported that experience of dog ownership is protectively linked to reduced incident frailty among Japanese seniors [9, 10], partly because dog owners have higher physical activity levels and social functioning than non-owners [11]. Building on this research, we now test the hypothesis that dog (and perhaps cat) ownership is linked to reduced risk of disability and/or mortality. We also hypothesize that regular exercise and close social relationships are moderating variables that may enhance the effects of dog/cat ownership in reducing the risk of disability and/or mortality.

There is already a substantial epidemiological recent literature on the potential health benefits of pet ownership. Ownership has been reported as being predictive of reduced mortality [12], improved cumulative survival rates [13], fewest doctor visits [14], and greater capacity to maintain ordinary activities of daily living (ADL) [15]. It is important to record, however, that these findings have been contested, with some large scale studies and literature reviews reporting no relationship between pet ownership and objectively measured health outcomes [16–18]. The value of the present study, it is submitted, lies in investigating the link between pet ownership and the highly reliable, well validated assessments of disability provided by the Japanese long-term care insurance system and of deaths provided by local registries. Further, we had the opportunity to conduct a study with a prospective design in which a large sample of older people provided baseline health measures. Implementing this design, we were able to estimate the effects of dog/cat ownership on the risk of subsequent disability and all-cause mortality risk, net of baseline health. It is hoped that the study yields new insights into health promotion for older adults; insights which may be of particular value during the current COVID-19 crisis in which restrictions have been imposed on face-to-face communication between people.

## Methods

### Participants

Data for the study were collected as part of a community-wide intervention trial (the Ota Genki Senior Project), which was launched in 2016. The details of the study design have been previously reported [19–21]. Briefly, 15500 residents aged 65–84 years—approximately 10% of the older population of Ota City, Tokyo Japan—were selected by using stratified and random sampling strategies in all 18 districts. All participants were physically and cognitively independent, defined as absence of long-term care insurance certification. In June 2016 we mailed a self-administered questionnaire to 15500 older residents as a baseline survey; 11925 questionnaires were returned (response rate 76.9%). The follow-up period was approximately 3.5 years.

To be eligible for the study, individuals had to complete a questionnaire on their experience of dog/cat ownership. Complete data were received from 11233 participants (rate of valid responses, 72.5%) [11]. All data collection in the Ota Genki Senior Project was carried out in accordance with the relevant guidelines of the Ethical Committee of the Tokyo Metropolitan Institute of Gerontology. We adhered strictly to the Declaration of Helsinki. This study was approved by the Ethical Committee of the Tokyo Metropolitan Institute of Gerontology. A statement attached to the questionnaire explained the purpose of the survey, the voluntary nature of participation, and promised anonymity in the analysis. Returning the questionnaire was taken to indicate consent to participate.

### Definition of dog/cat ownership

Participants were asked if they lived with a pet (current, past, or never). Those with current or past pet experience were asked about the pet species in the household (dog, cat, or other). These responses were used to classify dog ownership and cat ownership as current, past, or never [9, 11].

### Disability and all-cause mortality

Disability was determined by the Japanese long-term care insurance (LTCI) system [22]. Assessment is mandatory for every Japanese person aged 40 years and older. The certification processes include assessment of functional disability and cognitive disability. In this study the onset of disability was defined as new certification by the LTCI service [23]. We also examined local registries to ascertain deaths from any cause, and linked these data with Japanese national vital statistics [24, 25].

Because of the spread of COVID-19 in Japan in 2020, we selected the period of June 2016 to January 2020 as our follow-up period [26]. This avoided any overlap with disability or deaths that were due to Covid. In the end, of the 11233 initial participants, mortality data were available for 11228 participants (99.96%), and disability data for 11015 participants (98.06%).

### Covariates included in equations as ‘controls’

The covariates included in our estimation equations include sociodemographic characteristics: sex, age, household size, marital status, educational attainment, equivalent income, history of chronic diseases, history of hospitalization during the past year, alcohol drinking, smoking status, food variety, frailty status, exercise habits, interaction with neighbors, frequency of going outdoors, self-rated health, and a Geriatric Depression Scale [GDS]-5 score.

The chronic diseases that were evaluated included clinically relevant medical conditions, namely, hypertension, hyperlipidemia, heart disease, stroke, diabetes mellitus, lung respiratory disease, and cancer. For each of these conditions, participants were asked if they had received a physician diagnosis (yes or no). Food variety was assessed by dietary variety score, which was based on weekly consumption of 10 food items (meat, fish/shellfish, eggs, milk, soybean products, green/yellow vegetables, potatoes, fruit, seaweed, and fats/oils). The dietary variety score ranges from 0 to 10 points, with high scores indicating greater food variety [27]. Frailty status was assessed by a modified version of the Kaigo-Yobo Checklist. Scores range from 0 to 15, with a score higher than 4 being defined as ‘frail’ [28]. Participants were asked about types of exercise they engaged in more than once per week: no-exercise, walking, running, muscle training, stretching, swimming, cycling, yogic exercises, and other. Participants were then classified as having a regular exercise habit or no regular exercise habit. Interaction with neighbors was assessed dichotomously: high social interaction (close relationship and conversation level) and low social interaction (exchange of greetings only or no social contact).

### Statistical analysis: Logistic regression analysis

The primary statistical technique used to analyze the survey data is binomial logistic regression. Using this technique, we assess the net effects of dog/cat ownership on incident disability and all-cause mortality, controlling for sociodemographic variables, baseline health, and the length of the follow-up period. Logistic regression analysis is a parametric technique, but does not require a linear relationship between independent and dependent variables, nor do estimated residuals need to be normally distributed.

Potential confounders of the relationships between dog/cat ownership and disability and mortality were evaluated for collinearity. The socio-demographic covariates included in our final equations were: sex, age, household size, educational attainment, equivalent income, plus the 18 administrative districts in which respondents lived. The health measures included were: history of hypertension, heart disease, stroke, diabetes mellitus, lung respiratory disease, cancer, alcohol consumption, smoking status, food variety, frailty status, and depressive mood (GDS-5). Finally, we examined the combined effects of dog/cat ownership and exercise and/or social interaction with neighbors on incident disability and/or all-cause mortality, after controlling for potential confounders.

## Results

### Dog/cat ownership and sample characteristics

The mean (SD and range) age of participants was 74.2 (5.4 and 65–84) years, with 51.5% being women. At baseline 13.8% (n = 1545) of the sample were current dog or cat owners, 29.5% (n = 3311) were past owners, and 56.8% (n = 6377) had never been owners. Dog ownership was somewhat more prevalent than cat ownership: 8.6% (n = 963) were currently dog owners, 22.6% (n = 2540) were past owners, and 68.8% (n = 7730) never had a dog. The corresponding figures for cat ownership were 6.3% (n = 706), 11.1% (n = 1242), and 82.7% (n = 9285). As compared with those who had never owned a dog or cat, participants who were current owners were disproportionately women, younger, married or living with a partner, had more formal education, higher equivalent incomes, were more likely to drink alcohol and smoke, had a lower rate of frailty status, took more exercise, went outdoors more, and had closer relationships with neighbors (Table 1).

**Table 1:** Baseline demographic and health characteristics among community-dwelling older Japanese with and without experience of dog/cat ownership.

Variable	Experience of dog/cat ownership				
	Current (n = 1545, 13.8%)	Past (n = 3311, 29.5%)	Never (n = 6377, 56.8%)	Total (n = 11233)	P-Value
Sex (female)	52.7	53.3	50.4	51.5	.016
Age, years (%)					< .001
65–74	59.4	46.3	45.6	47.7	
75–84	40.6	53.7	54.4	52.3	
Household size (%)					< .001
Living alone	9.1	18.5	23.8	22.2	
Living together	89.3	79.3	74.1	77.7	
Missing	1.6	2.2	2.1	2.1	
Marital status (%)					< .001
Married	72.8	67.2	63.3	65.8	
Divorced, Widowed, Single	25.6	30.8	34.6	32.2	
Missing	1.6	2.0	2.1	2.0	
Educational attainment (%)					< .001
Elementary school, Middle school, Others	21.0	21.7	29.8	26.3	
High school	36.4	36.5	39.0	37.9	
College, university, or graduate school	41.2	40.4	29.5	34.3	
Missing	1.4	1.4	1.7	1.5	
Equivalent income (%)					< .001
<1,000,000 yen	5.6	6.4	7.3	6.8	
1,000,000 yen– 2,500,000 yen	26.7	28.6	33.3	31.0	
2,500,000 yen– 4,000,000 yen	23.0	23.8	24.5	24.1	
≥4,000,000 yen	24.2	21.7	15.3	18.4	
Unknown	15.9	13.7	12.9	13.5	
Missing	4.7	5.8	6.6	6.1	
History of hypertension (%)					.743
Yes	52.1	51.9	52.2	52.1	
No	43.9	44.2	43.4	43.7	
Missing	3.9	3.9	4.4	4.2	
History of hyperlipidemia (%)					.332
Yes	41.9	39.7	39.3	39.8	
No	52.1	54.7	54.6	54.3	
Missing	6.0	5.6	6.1	5.9	
History of heart disease (%)					.470
Yes	19.9	21.7	20.4	20.7	
No	74.1	73.0	73.7	73.6	
Missing	6.0	5.4	5.9	5.7	
History of stroke (%)					.689
Yes	8.0	7.4	7.0	7.3	
No	86.0	87.1	87.2	87.0	
Missing	6.0	5.5	5.8	5.8	
History of diabetes mellitus (%)					.983
Yes	17.8	17.5	17.9	17.7	
No	76.9	77.3	77.1	77.1	
Missing	5.3	5.2	5.1	5.1	
History of lung respiratory disease (%)					.153
Yes	13.5	15.6	13.8	11.9	
No	80.8	79.2	80.7	80.3	
Missing	5.8	5.3	5.5	5.4	
History of cancer (%)					.329
Yes	17.2	16.2	15.2	15.8	
No	77.3	78.0	79.2	78.6	
Missing	5.5	5.8	5.6	5.6	
Alcohol drinking status (%)					.024
Current	56.7	55.5	53.3	54.4	
Past	6.7	8.5	8.2	8.1	
Never	35.7	35.0	37.2	36.4	
Missing	0.9	0.9	1.3	1.1	
Smoking status (%)					.002
Current	14.4	11.7	12.2	12.4	
Past	33.5	34.1	31.0	32.3	
Never	50.6	52.7	54.9	53.7	
Missing	1.4	1.5	1.8	1.7	
Food variety (%)					.080
≥4 points	35.8	38.3	36.4	36.9	
0–3 points	56.1	52.9	53.9	53.9	
Missing	8.1	8.9	9.6	9.2	
Frailty (%)	22.1	22.4	24.7	23.7	.011
Exercise habit (%)					.015
Yes	73.3	74.1	71.4	72.5	
No	25.4	23.9	26.6	25.6	
Missing	1.3	2.0	2.0	1.9	
Interaction with neighbors (%)					< .001
Significant relationship	24.1	25.1	22.0	23.2	
Conversation	40.1	38.2	36.1	37.3	
Exchange of greetings only, No social contact	35.5	36.4	41.5	39.2	
Missing	0.3	0.3	0.4	0.3	
Frequency of going outdoors (%)					< .001
At least once a day	78.3	72.3	73.4	73.7	
Less than once every 2–3 days	20.8	26.8	25.2	25.1	
Missing	1.0	0.8	1.4	1.2	
Self-rated health (%)					.005
Excellent to good	77.8	77.3	74.5	75.8	
Fair to poor	15.5	16.6	18.8	17.7	
Missing	6.7	6.2	6.7	6.5	
GDS-5 (%)					.280
≥2 points	31.3	31.7	32.7	32.2	
0–1 point	62.8	61.8	60.4	61.2	
Missing	5.8	6.5	6.9	6.7	

P-values were calculated using a two-tailed Pearson’s chi-square test. BMI = body mass index. GDS = Geriatric Depression Scale.

### Onset of disability and death

During the 3.5 year follow-up period, 17.1% (1882 out of 11015 participants) were identified as suffering onset of disability, and 5.2% (589 out of 11228 participants) died. The raw (unadjusted) onset of disability rate of 13.1% for current dog owners was lower than for past owners (16.8%) and ‘never’ owners (17.7%); these differences being statistically significant at the 0.01 level. The unadjusted death rate of current dog owners at 4.0% was lower than the sample average of 5.2%; this was not statistically significant at the 0.05 level.

### Do dog/cat owners record lower rates of disability and death, net of confounding effects?

The results of a series of logistic regression analyses relating to the onset of disability are given in Table 2. Similar analyses relating to all-cause mortality are presented in Table 3. The outcome variables in both tables are odds ratios: the odds ratio of the onset of disability in Table 2, and the odds ratio of death in Table 3. The reference groups in both tables are study participants who were ‘never’ dog/cat owners. Their odds ratio is fixed at 1.0. So an odds ratio for dog/cat owners of less than 1.0 means that they were at lower risk of an adverse outcome than ‘never’ owners, whereas an odd ratio over 1.0 means higher risk. Model 1 in both Tables 2 and 3 reports odds ratios, controlling only for socio-demographic variables. Model 2 in both tables reports odds ratios showing the net effects of dog/cat ownership adjusting for both socio-demographic variables and health at baseline.

**Table 2:** Logistic regression models estimating the net effects of dog and cat ownership on the onset of disability. Model 1 includes controls for socio-demographic variables. Model 2 also adds controls for health at baseline.

		Total (n = 11015)	
	Incident disability	Model 1, OR (95%CI)	Model 2, OR (95%CI)
Dog/Cat ownership			
Never §	1118/6244 (17.9%)	1	1
Past	556/3256 (17.1%)	0.91 (0.75–1.10)	0.88 (0.73–1.08)
Current	208/1515 (13.7%)	0.72 (0.54–0.96) *	0.71 (0.53–0.95) *
Dog ownership			
Never §	1339/7575 (17.7%)	1	1
Past	419/2493 (16.8%)	0.87 (0.70–1.07)	0.84 (0.68–1.03)
Current	124/947 (13.1%)	**0.54 (0.37–0.78)** **	**0.54 (0.38–0.79)** **
Cat ownership			
Never §	1564/9102 (17.2%)	1	1
Past	216/1222 (17.7%)	1.00 (0.76–1.30)	0.98 (0.75–1.29)
Current	102/691 (14.8%)	1.08 (0.75–1.54)	(0.74–1.53)

*p<0.05

**p<0.01.

OR, odds ratio; CI, confidence interval

§ reference group.

Model 1 includes controls for socio-demographic variables; sex, age, household size, educational attainment, equivalent income, and administrative districts.

Model 2 adds controls for health measures; history of hypertension, heart disease, stroke, diabetes mellitus, lung respiratory disease, and cancer, alcohol drinking and smoking status, food variety, frailty, Geriatric Depression Scale, and follow-up period.

**Table 3:** Logistic regression models estimating the net effects of dog and cat ownership on all-cause mortality. Model 1 controls for socio-demographic variables.; Model 2 also adds controls for health at baseline.

		Total (n = 11228)	
	Incident all-cause mortality	Model-1, OR (95%CI)	Model-2, OR (95%CI)
Dog/Cat ownership			
Never §	355/6375 (5.6%)	1	1
Past	165/3308 (5.0%)	0.95 (0.74–1.24)	0.93 (0.71–1.21)
Current	69/1545 (4.5%)	0.78 (0.54–1.12)	0.80 (0.55–1.16)
Dog ownership			
Never §	432/7727 (5.6%)	1	1
Past	118/2538 (4.6%)	0.83 (0.63–1.11)	0.78 (0.58–1.05)
Current	39/963 (4.0%)	0.66 (0.42–1.05)	0.70 (0.44–1.12)
Cat ownership			
Never §	484/9281 (5.2%)	1	1
Past	68/1241 (5.5%)	1.15 (0.81–1.63)	1.16 (0.81–1.65)
Current	37/706 (5.2%)	1.10 (0.69–1.75)	1.12 (0.69–1.81)

*p<0.05.

OR, odds ratio; CI, confidence interval

§ reference group.

Model 1 includes controls for socio-demographic variables; sex, age, household size, educational attainment, equivalent income, and administrative districts.

Model 2 adds controls for health measures; history of hypertension, heart disease, stroke, diabetes mellitus, lung respiratory disease, and cancer, alcohol drinking and smoking status, food variety, frailty, Geriatric Depression Scale, and follow-up period.

We begin with what we regard as our most important result. In Model 1, older adults who currently own a dog are estimated to be at much lower risk of disability than those who are not owners (Table 2, S1 Table). They have approximately half the risk of disability (odds ratio = 0.54, 95% CI:0.37–0.78) as those who never had a dog. Adjusting for sociodemographic variables and health makes virtually no difference to this result. The odds ratio remains the same at 0.54 with the confidence interval just slightly increasing (CI:0.38–0.79).

Cat ownership appears to have no effect on the onset of disability (Table 2, S1 Table). Neither current nor past owners are at lower risk of disability than those who never owned a cat. Adjusting for sociodemographic variables and health makes virtually no difference to these results.

### Combined effects: Exercise and social interaction with neighbors

**Fig 1:**
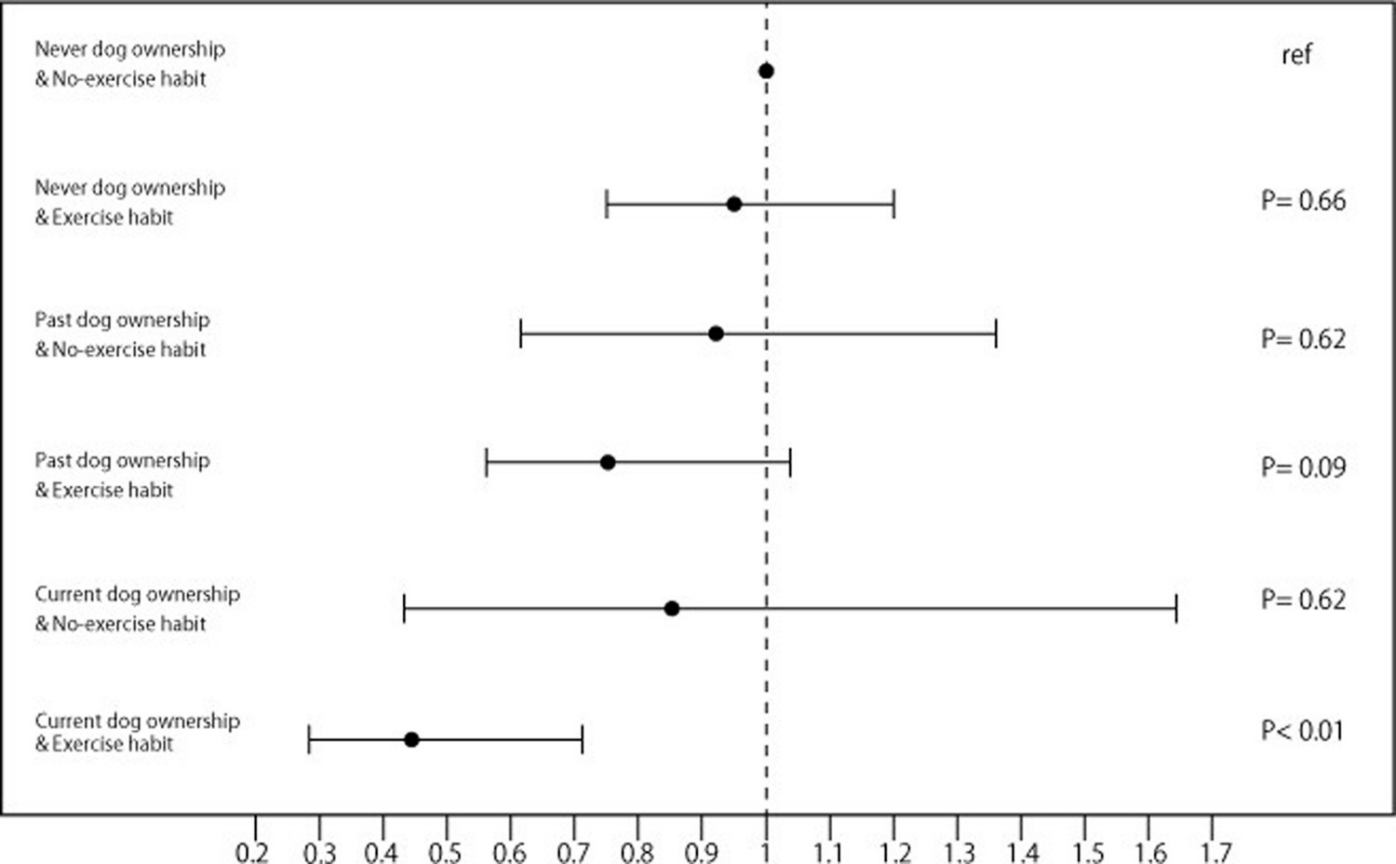
Odds ratios of dog ownership and habitual exercise with incident disability. Adjusted for socio-demographic variables (sex, age, household size, years of education, equivalent income, and administrative district), health measures (history of hypertension, heart disease, stroke, diabetes mellitus, lung respiratory disease, and cancer, alcohol drinking and smoking status, food variety, frailty, Geriatric Depression Scale), and follow-up period.

**Fig 2:**
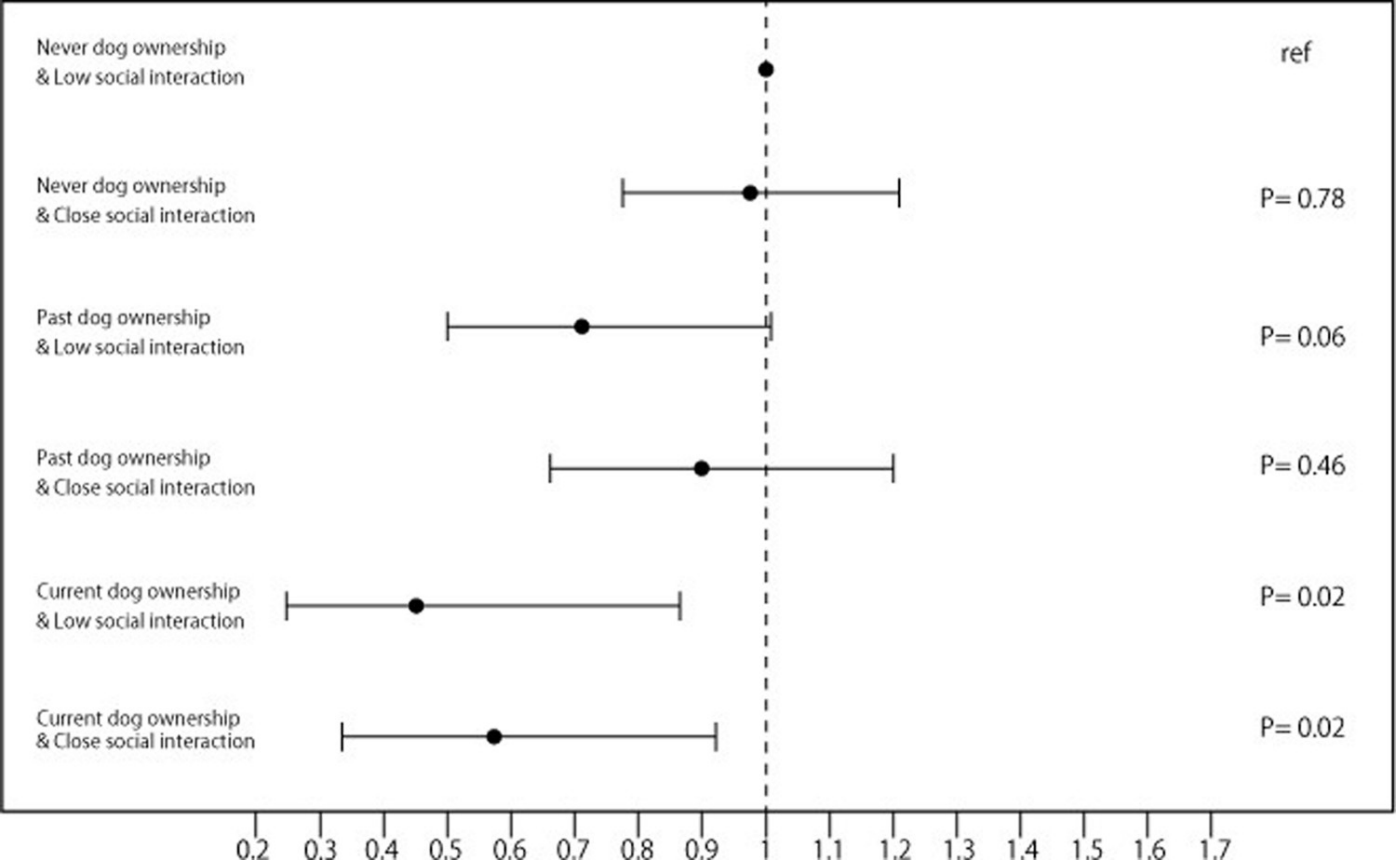
Odds ratios of dog ownership and social relationship with incident disability. Adjusted for socio-demographic variables (sex, age, household size, years of education, equivalent income, and administrative district), health measures (history of hypertension, heart disease, stroke, diabetes mellitus, lung respiratory disease, and cancer, alcohol drinking and smoking status, food variety, frailty, Geriatric Depression Scale), and follow-up period.

We also attempted to estimate the combined effects on disability onset of dog ownership, exercise habits and social interaction (Figs 1 and 2). In our modelling we viewed these as moderating variables that might further reduce the risk of disability. First, exercise habits: we categorized participants into 6 groups, each described by a combination: current dog ownership with a regular exercise habit (n = 741, 6.7%), current dog ownership with no-exercise habit (n = 213, 1.9%), past dog ownership with regular exercise (n = 1900, 17.2%), past dog ownership with no-exercise habit (n = 597, 5.4%), ‘never’ dog ownership with exercise habit (n = 5498, 49.9%), and ‘never’ dog ownership with no-exercise habit (n = 2069, 18.8%). A crucial result here is that the odds ratio of disability for current dog owners who do regular exercise is 0.44 (95% CI:0.28–0.71), after controlling for socio-economic variables and health measures (Fig 1). This new result indicates that older dog owners who also take regular exercise are at significantly lower risk.

A similar analysis was undertaken to estimate possible interactions between dog ownership and social relationships with neighbors. The 6 groups are as follows: current dog ownership with close social relationships (n = 635, 5.7%), current dog ownership with low social relationships (n = 325, 2.9%), past dog ownership with close social relationships (n = 1595, 14.2%), past dog ownership with low social relationships (n = 938, 8.4%), ‘never’ dog ownership with close social relationships (n = 4566, 40.8%), and ‘never’ dog ownership with low social relationships (n = 3136, 28.0%). The results indicate that, net of ‘controls’, social relationships with neighbours have no additional effect for dog owners in reducing the odds of disability onset (Fig 2).

### All-cause mortality

The results in Table 3 and S2 Table indicate that dog and cat ownership do not reduce the risk of all-cause mortality in the total sample. The odds ratios of current owners do not differ significantly from those of past or ‘never’ owners. Only older men who were past owners of a dog are estimated to be at a statistically significant lower risk of mortality (odds ratio = 0.65, 95% CI:0.45–0.96). This could be just a statistical anomaly, given that the risk to current dog owners are not estimated to be significantly reduced.

## Discussion

The main findings of this large scale, prospective study of community-dwelling older Japanese are that older adults who are dog owners are at reduced risk of disability compared with non-owners. The risk is further reduced by taking regular exercise. these findings hold, net of the potentially confounding effects of socio-demographic health at baseline.

The benefits of regular exercise are worth underlining. ^27, 28^Most dog owners take their pet for regular walks; indeed, dog owners are four times more likely than non-owners to meet recommended physical activity guidelines [29]. Dog walking is a moderate-intensity physical activity that appears to have a protective effect in reducing the risk of disability onset through decreased frailty risk. It can be an important component of successful aging.

In previous research it was reported that social activities such as engagement in cultural activities and community groups are associated with a lower risk of developing disability [30]. However, in this study we find that close neighborhood relationships appear to make no additional contribution to preventing disability, over and above dog ownership. These apparently disparate findings may not contradictory; a reasonable interpretation, perhaps, is that the companionship of a pet dog can partly compensate for limited human interaction. Again, the implication is that dog ownership may play a role in successful aging, perhaps especially at a time when face-to-face interactions are restricted due to the COVID outbreak.

A negative result in this study is that no link was found between dog or cat ownership and all-cause mortality. Several previous studies reported that dog ownership is associated with both lower cardiovascular and all-cause mortality [12, 13]. Contrary to these results, Ding et al’s comprehensive review found no evidence for these linkages [15]. Clearly, our results tend to support the negative case, but explanations for apparently contradictory results need to be found, and require further research.

This study has some strengths that warrant mention. First, our large sample of community-dwelling older Japanese enabled analysis based on classifying dog and cat owners into sub-groups of current, past and ‘never’ owners. We were also able to undertake analysis of interaction effects between pet ownership, regular exercise and social relationships with neighbors, while still controlling for a fairly comprehensive set of socio-demographic and health variables. Our detailed analysis indicates that current dog ownership is beneficial for older adults, substantially reducing their risk of disability onset. (sentence deleted here–too much repetition).

In future work it will be important to consider the psychological pathways that may link dog ownership to reduced disability onset. A recent study reported that dog ownership improves the psychological well-being of socially isolated older adults [31]. It is known that psychological well-being is inversely related to the onset of frailty [32]. In short, further research is needed to explore links between dog ownership, psychological variables, frailty and disability. It will also be important to assess whether linkages found in Japan are replicated in Western and other Asian countries.

In summary, this prospective study is the first, so far as we are aware, to indicate that dog ownership may well be protective against the onset of disability in older adults. The daily care, companionship and exercise of a pet dog may be recommended as a component of health promotion policy, and may have an important role to play in successful aging.

## Supporting information

S1 Table Associations of dog and cat ownership with the onset of disability. *p<0.05, **p<0.01. OR, odds ratio; CI, confidence interval; § reference group. Model 1 includes controls for socio-demographic variables; sex, age, household size, educational attainment, equivalent income, and administrative districts. Model 2 adds controls for health measures; history of hypertension, heart disease, stroke, diabetes mellitus, lung respiratory disease, and cancer, alcohol drinking and smoking status, food variety, frailty, Geriatric Depression Scale, and follow-up period.
(DOCX)

S2 Table Associations of dog and cat ownership with all-cause mortality. *p<0.05, **p<0.01. OR, odds ratio; CI, confidence interval; § reference group. Model 1 includes controls for socio-demographic variables; sex, age, household size, educational attainment, equivalent income, and administrative districts. Model 2 adds controls for health measures; history of hypertension, heart disease, stroke, diabetes mellitus, lung respiratory disease, and cancer, alcohol drinking and smoking status, food variety, frailty, Geriatric Depression Scale, and follow-up period.
(DOCX)
