# Correction: Evidence that dog ownership protects against the onset of disability in an older community-dwelling Japanese population

**DOI:** 10.1371/journal.pone.0314379

**Published:** 2024-11-20

**Authors:** The PLOS ONE Staff

In the Disability and all-cause mortality subsection of the Methods, there is an error in the first sentence of the first paragraph. The correct sentence is: Disability was determined by the Japanese long-term care insurance (LTCI) system [22] and/or death [23].

In Table 2, the odds ratio under Model 2 column for current cat ownership is missing. It should have been 1.06. Please view the correct Table 2 below.

**Table 2:** Logistic regression models estimating the net effects of dog and cat ownership on the onset of disability. Model 1 includes controls for socio-demographic variables. Model 2 also adds controls for health at baseline.

		Total (n = 11015)	
	Incident disability	Model 1, OR (95%CI)	Model 2, OR (95%CI)
Dog/Cat ownership			
Never §	1118/6244 (17.9%)	1	1
Past	556/3256 (17.1%)	0.91 (0.75–1.10)	0.88 (0.73–1.08)
Current	208/1515 (13.7%)	0.72 (0.54–0.96) *	0.71 (0.53–0.95) *
Dog ownership			
Never §	1339/7575 (17.7%)	1	1
Past	419/2493 (16.8%)	0.87 (0.70–1.07)	0.84 (0.68–1.03)
Current	124/947 (13.1%)	0.54 (0.37–0.78) **	0.54 (0.38–0.79) **
Cat ownership			
Never §	1564/9102 (17.2%)	1	1
Past	216/1222 (17.7%)	1.00 (0.76–1.30)	0.98 (0.75–1.29)
Current	102/691 (14.8%)	1.08 (0.75–1.54)	1.06 (0.74–1.53)

*p<0.05,

**p<0.01

OR, odds ratio; CI, confidence interval; § reference group.

Model 1 includes controls for socio-demographic variables; sex, age, household size, educational attainment, equivalent income, and administrative districts.

Model 2 adds controls for health measures; history of hypertension, heart disease, stroke, diabetes mellitus, lung respiratory disease, and cancer, alcohol drinking and smoking status, food variety, frailty, Geriatric Depression Scale, and follow-up period.

The publisher apologizes for the errors.
