# Supplementary material for: Evidence that dog ownership protects against the onset of disability in an older community-dwelling Japanese population
Source: PLoS One. 2022 Feb 23;17(2):e0263791. doi: 10.1371/journal.pone.0263791 (PMC8865647; doi:10.1371/journal.pone.0263791)
Supplement: S1 Table — *p<0.05, **p<0.01. OR, odds ratio; CI, confidence interval; § reference group. Model 1 includes controls for socio-demographic variables; sex, age, household size, educational attainment, equivalent income, and administrative districts. Model 2 adds controls for health measures; history of hypertension, heart disease, stroke, diabetes mellitus, lung respiratory disease, and cancer, alcohol drinking and smoking status, food variety, frailty, Geriatric Depression Scale, and follow-up period. (DOCX) [file pone.0263791.s001.docx]

　Supplemental Table 1.

Associations of Dog and Cat Ownership with the Onset of Disability.

|  | Model 1, OR (95%CI) | Model 2, OR (95%CI) | Model 1, OR (95%CI) | Model 2, OR (95%CI) | Model 1, OR (95%CI) | Model 2, OR (95%CI) |
| --- | --- | --- | --- | --- | --- | --- |
| Dog/Cat ownership |  |  |  |  |  |  |
| Never § | 1 | 1 |  |  |  |  |
| Past | 0.91 (0.75-1.10) | 0.88 (0.73-1.08) |  |  |  |  |
| Current | 0.72 (0.54-0.96) * | 0.71 (0.53-0.95) * |  |  |  |  |
| Dog ownership |  |  |  |  |  |  |
| Never § |  |  | 1 | 1 |  |  |
| Past |  |  | 0.87 (0.70-1.07) | 0.84 (0.68-1.03) |  |  |
| Current |  |  | **0.54 (0.37-0.78) **** | **0.54 (0.38-0.79) **** |  |  |
| Cat ownership |  |  |  |  |  |  |
| Never § |  |  |  |  | 1 | 1 |
| Past |  |  |  |  | 1.00 (0.76-1.30) | 0.98 (0.75-1.29) |
| Current |  |  |  |  | 1.08 (0.75-1.54) | 1.06 (0.74-1.53) |
| Sex (male vs female) | 1.17 (0.98-1.40) | 0.96 (0.77-1.21) | 1.17 (0.98-1.39) | 0.96 (0.76-1.21) | 1.18 (0.99-1.41) | 0.99 (0.78-1.24) |
| Age | 1.15 (1.13-1.18) ** | 1.15 (1.13-1.17) ** | 1.15 (1.13-1.18) ** | 1.15 (1.13-1.17) ** | 1.16 (1.14-1.18) ** | 1.15 (1.13-1.17) ** |
| Household size (living together vs missing) | 1.50 (0.77-2.94) | 1.70 (0.85-3.40) | 1.50 (0.77-2.92) | 1.69 (0.85-3.38) | 1.48 (0.75-2.90) | 1.66 (0.83-3.34) |
| Educational attainment (College, university, or graduate school vs missing) | 0.78 (0.39-1.56) | 0.90 (0.44-1.83) | 0.79 (0.40-1.59) | 0.92 (0.45-1.85) | 0.77 (0.39-1.55) | 0.89 (0.44-1.81) |
| Equivalent income (≥4,000,000 yen vs missing) | 0.78 (0.39-1.56) | 0.70 (0.45-1.09) | 0.61 (0.39-0.94) * | 0.70 (0.45-1.09) | 0.60 (0.39-0.93) * | 0.69 (0.44-1.07) |
| Administrative districts | 1.01 (0.99-1.03) | 1.01 (0.99-1.03) | 1.01 (0.99-1.03) | 1.01 (0.99-1.03) | 1.01 (0.99-1.03) | 1.01 (0.99-1.03) |
| History of hypertension (no vs yes) |  | 0.94 (0.77-1.12) |  | 0.93 (0.77-1.12) |  | 0.93 (0.78-1.12) |
| History of heart disease (no vs yes) |  | 0.92 (0.75-1.15) |  | 0.92 (0.74-1.14) |  | 0.93 (0.75-1.15) |
| History of stroke (no vs yes) |  | 0.99 (0.72-1.38) |  | 1.00 (0.72-1.39) |  | 1.01 (0.73-1.40) |
| History of diabetes mellitus (no vs yes) |  | 0.72 (0.57-0.89) ** |  | 0.72 (0.57-0.89) ** |  | 0.72 (0.57-0.89) ** |
| History of lung respiratory disease (no vs yes) |  | 0.79 (0.63-1.00) * |  | 0.79 (0.62-0.99) * |  | 0.79 (0.62-0.99) * |
| History of cancer (no vs yes) |  | 0.71 (0.57-0.89) ** |  | 0.71 (0.57-0.89) ** |  | 0.71 (0.57-0.89) ** |
| Alcohol drinking status (current vs never) |  | 0.78 (0.64-0.96) * |  | 0.78 (0.64-0.96) * |  | 0.77 (0.63-0.95) * |
| Smoking status (current vs never) |  | 1.47 (1.08-1.99) * |  | 1.47 (1.08-1.99) * |  | 1.45 (1.07-1.96) * |
| Food variety (0-3 points vs ≥4 points) |  | 0.89 (0.73-1.08) |  | 0.89 (0.73-1.08) |  | 0.88 (0.73-1.07) |
| Frailty (no vs yes) |  | 0.63 (0.51-0.76) ** |  | 0.63 (0.52-0.77) ** |  | 0.63 (0.51-0.76) ** |
| Geriatric Depression Scale (0-1 point vs ≥2 points |  | 0.86 (0.71-1.05) |  | 0.86 (0.71-1.05) |  | 0.86 (0.71-1.05) |
| Follow-up period |  | 0.78 (0.77-0.79) ** |  | 0.78 (0.77-0.79) ** |  | 0.78 (0.78-0.79) ** |

*p<0.05, **p<0.01

OR, odds ratio; CI, confidence interval; § reference group.
