# Supplementary material for: Evidence that dog ownership protects against the onset of disability in an older community-dwelling Japanese population
Source: PLoS One. 2022 Feb 23;17(2):e0263791. doi: 10.1371/journal.pone.0263791 (PMC8865647; doi:10.1371/journal.pone.0263791)
Supplement: S2 Table — *p<0.05, **p<0.01. OR, odds ratio; CI, confidence interval; § reference group. Model 1 includes controls for socio-demographic variables; sex, age, household size, educational attainment, equivalent income, and administrative districts. Model 2 adds controls for health measures; history of hypertension, heart disease, stroke, diabetes mellitus, lung respiratory disease, and cancer, alcohol drinking and smoking status, food variety, frailty, Geriatric Depression Scale, and follow-up period. (DOCX) [file pone.0263791.s002.docx]

Supplemental Table 2.

Associations of Dog and Cat Ownership with All-Cause Mortality.

|  | Model 1, OR (95%CI) | Model 2, OR (95%CI) | Model 1, OR (95%CI) | Model 2, OR (95%CI) | Model 1, OR (95%CI) | Model 2, OR (95%CI) |
| --- | --- | --- | --- | --- | --- | --- |
| Dog/Cat ownership |  |  |  |  |  |  |
| Never § | 1 | 1 |  |  |  |  |
| Past | 0.95 (0.74-1.24) | 0.93 (0.71-1.21) |  |  |  |  |
| Current | 0.78 (0.54-1.12) | 0.80 (0.55-1.16) |  |  |  |  |
| Dog ownership |  |  |  |  |  |  |
| Never § |  |  | 1 | 1 |  |  |
| Past |  |  | 0.83 (0.63-1.11) | 0.78 (0.58-1.05) |  |  |
| Current |  |  | 0.66 (0.42-1.05) | 0.70 (0.44-1.12) |  |  |
| Cat ownership |  |  |  |  |  |  |
| Never § |  |  |  |  | 1 | 1 |
| Past |  |  |  |  | 1.15 (0.81-1.63) | 1.16 (0.81-1.65) |
| Current |  |  |  |  | 1.10 (0.69-1.75) | 1.12 (0.69-1.81) |
| Sex (male vs female) | 2.99 (2.33-3.84) ** | 2.31 (1.68-3.19) ** | 2.99 (2.33-3.85) ** | 2.30 (1.67-3.17) ** | 3.03 (2.36-3.89) ** | 2.36 (1.72-3.26) ** |
| Age | 1.10 (1.08-1.13) ** | 1.10 (1.07-1.12) ** | 1.10 (1.08-1.13) ** | 1.10 (1.07-1.12) ** | 1.10 (1.08-1.13) ** | 1.10 (1.07-1.12) ** |
| Household size (living together vs missing) | 3.58 (1.36-9.40) ** | 3.46 (1.32-9.09) ** | 3.62 (1.38-9.51) ** | 3.48 (1.33-9.12) ** | 3.56 (1.36-9.35) ** | 3.45 (1.32-9.05) ** |
| Educational attainment (College, university, or graduate school vs missing) | 0.92 (0.36-2.36) | 0.91 (0.36-2.30) | 0.95 (0.37-2.44) | 0.93 (0.37-2.36) | 0.91 (0.36-2.33) | 0.89 (0.35-2.26) |
| Equivalent income (≥4,000,000 yen vs missing) | 0.57 (0.32-1.02) | 0.68 (0.38-1.24) | 0.57 (0.32-1.02) | 0.68 (0.38-1.24) | 0.56 (0.31-1.00) | 0.67 (0.37-1.22) |
| Administrative districts | 1.02 (0.99-1.04) | 1.01 (0.98-1.04) | 1.01 (0.99-1.04) | 1.01 (0.98-1.04) | 1.02 (0.99-1.04) | 1.01 (0.98-1.04) |
| History of hypertension (no vs yes) |  | 1.10 (0.86-1.40) |  | 1.10 (0.86-1.40) |  | 1.10 (0.86-1.41) |
| History of heart disease (no vs yes) |  | 0.81 (0.62-1.07) |  | 0.81 (0.62-1.06) |  | 0.81 (0.62-1.07) |
| History of stroke (no vs yes) |  | 1.31 (0.88-1.97) |  | 1.31 (0.87-1.96) |  | 1.33 (0.89-1.99) |
| History of diabetes mellitus (no vs yes) |  | 0.64 (0.49-0.84) ** |  | 0.64 (0.49-0.84) ** |  | 0.64 (0.49-0.84) ** |
| History of lung respiratory disease (no vs yes) |  | 0.68 (0.51-0.91) ** |  | 0.68 (0.51-0.90) ** |  | 0.69 (0.52-0.91) ** |
| History of cancer (no vs yes) |  | 0.50 (0.39-0.66) ** |  | 0.50 (0.38-0.65) ** |  | 0.51 (0.39-0.66) ** |
| Alcohol drinking status (current vs never) |  | 0.69 (0.52-0.91) ** |  | 0.69 (0.52-0.91) ** |  | 0.68 (0.51-0.90) ** |
| Smoking status (current vs never) |  | 1.78 (1.23-2.59) ** |  | 1.79 (1.24-2.60) ** |  | 1.78 (1.22-2.58) ** |
| Food variety (0-3 points vs ≥4 points) |  | 0.87 (0.67-1.13) |  | 0.87 (0.67-1.13) |  | 0.86 (0.66-1.11) |
| Frailty (no vs yes) |  | 0.53 (0.41-0.68) ** |  | 0.53 (0.41-0.68) ** |  | 0.52 (0.41-0.68) ** |
| Geriatric Depression Scale (0-1 point vs ≥2 points |  | 0.86 (0.67-1.12) |  | 0.86 (0.67-1.12) |  | 0.86 (0.67-1.12) |
| Follow-up period |  | 0.83 (0.82-0.84) ** |  | 0.83 (0.82-0.84) ** |  | 0.83 (0.82-0.84) ** |

*p<0.05, **p<0.01

OR, odds ratio; CI, confidence interval; § reference group.

Model 1 includes controls for socio-demographic variables; sex, age, household size, educational attainment, equivalent income, and administrative districts.

Model 2 adds controls for health measures; history of hypertension, heart disease, stroke, diabetes mellitus, lung respiratory disease, and cancer, alcohol drinking and smoking status, food variety, frailty, Geriatric Depression Scale, and follow-up period.
